# Nanomedicine‐Based Therapeutics for Myocardial Ischemic/Reperfusion Injury

**DOI:** 10.1002/adhm.202300161

**Published:** 2023-04-10

**Authors:** Xi Li, Wei Ou, Maodi Xie, Jing Yang, Qian Li, Tao Li

**Affiliations:** ^1^ Department of Anesthesiology Laboratory of Mitochondria and Metabolism National Clinical Research Center for Geriatrics West China Hospital of Sichuan University Chengdu 610041 P. R. China; ^2^ Department of Anesthesiology Nanchong Central Hospital Nanchong 637000 P. R. China

**Keywords:** antioxidation, myocardial ischemic/reperfusion injury, nanomedicines, reactive oxygen species

## Abstract

Myocardial ischemic/reperfusion (IR) injury is a global cardiovascular disease with high mortality and morbidity. Therapeutic interventions for myocardial ischemia involve restoring the occluded coronary artery. However, reactive oxygen species (ROS) inevitably impair the cardiomyocytes during the ischemic and reperfusion phases. Antioxidant therapy holds great promise against myocardial IR injury. The current therapeutic methodologies for ROS scavenging depend predominantly on administering antioxidants. Nevertheless, the intrinsic drawbacks of antioxidants limit their further clinical transformation. The use of nanoplatforms with versatile characteristics greatly benefits drug delivery in myocardial ischemic therapy. Nanoplatform‐mediated drug delivery significantly improves drug bioavailability, increases therapeutic index, and reduces systemic toxicity. Nanoplatforms can be specifically and reasonably designed to enhance molecule accumulation at the myocardial site. The present review initially summarizes the mechanism of ROS generation during the process of myocardial ischemia. The understanding of this phenomenon will facilitate the advancement of innovative therapeutic strategies against myocardial IR injury. The latest developments in nanomedicine for treating myocardial ischemic injury are then discussed. Finally, the current challenges and perspectives in antioxidant therapy for myocardial IR injury are addressed.

## Graphical abstract

Antioxidant enzymes are widely exploited to mediate cardiovascular disease therapies. This review summarizes the recent advancements in reactive oxygen species (ROS)‐based therapeutics for myocardial ischemic/reperfusion injury. The review describes the mechanisms that generate ROS during myocardial ischemia. It addresses the current challenges, perspectives, and future directions for the biomedical applications of these nanomedicine‐based ROS scavengers.

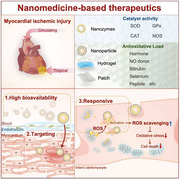

## 1 Introduction

Myocardial ischemic injury constitutes the universal leading cause of disability and death, which accounted for as much as 1.72% of the global population.^[^ ^1^, ^2^ ^]^ Myocardial infarction (MI) is a major cardiovascular disease characterized by irreversible damage to cardiac contractility, ultimately leading to death.^[^ ^3^ ^]^ Percutaneous coronary intervention and pharmaceutical drugs are commonly used to treat MI.^[^ ^4^ ^]^ These approaches significantly improve patient survival rates. However, blood restoration induces paradoxical cardiomyocyte death, termed ischemic/reperfusion (IR) injury. Reperfusion reportedly causes myocardial injury by bursting reactive oxygen species (ROS).^[^ ^5^, ^6^ ^]^ The overproduction of this ROS triggers cascades of inflammation reactions that consequently increase ROS generation and heart remodeling. Therefore, an effective strategy needs to be developed to preserve cardiomyocyte death and avoid the progression of heart remodeling.^[^ ^7^ ^]^ Great efforts are underway to develop antioxidants, such as enzymes and small molecule drugs, that scavenge ROS and treat myocardial ischemia.^[^ ^8^, ^9^ ^]^ However, poor pharmacokinetics and potential side effects limit the clinical applications of these antioxidants.

The emergence of nanotechnology has provided a promising opportunity for overcoming the challenges of these antioxidants. Nanomedicine is considered an appropriate strategy for drug delivery because of its improved pharmacokinetics, reduced systemic toxicity, and enhanced drug accumulation in targeted tissues.^[^ ^10^ ^]^ Various biomaterials, such as liposomes, microneedles, cardiac patches, and hydrogels, have been investigated to protect against cardiac ischemic/reperfusion injury by eliminating ROS.^[^ ^10^, ^11^ ^]^ Nanomaterials with ROS‐scavenging properties are specifically attractive for treating myocardial ischemia.^[^ ^12^, ^13^, ^14^ ^]^ Compared to traditional antioxidants, nano‐antioxidants (termed nanozymes) have unique advantages such as high catalytic stability, excellent ROS‐scavenging ability, and adjustable physicochemical properties. Thus, nanoparticles represent an attractive alternative approach to treating myocardial ischemia.^[^ ^15^, ^16^ ^]^ The efficiency of drug delivery is further improved by controllable structure modification. Interestingly, therapeutic molecule delivery can be targeted at disease sites with abnormal ROS levels.^[^ ^17^, ^18^, ^19^ ^]^ Based on these characteristics, ROS‐responsive nanomedicine has been reasonably designed to improve the cardiac therapeutic effect.

**Figure 1:**
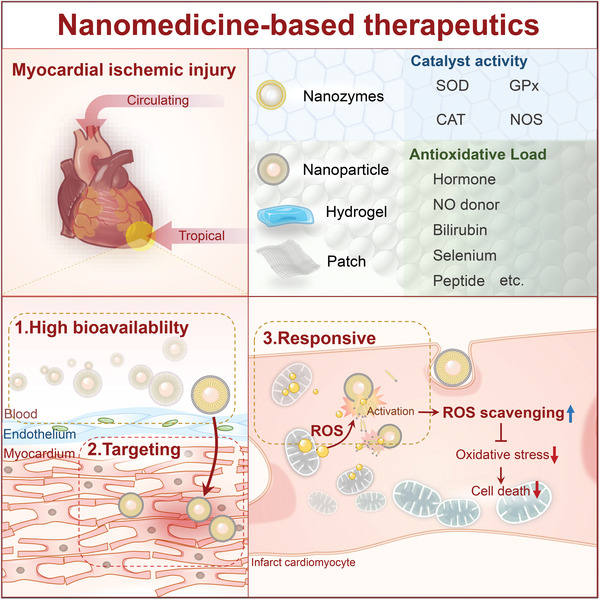
Schematic illustration of nanomedicine‐based therapeutics in myocardial ischemic disease.

Increasing numbers of biomaterials have been designed recently for efficient myocardial ischemia treatment. A systematic summary is essential for developing myocardial ischemic therapeutic strategies in the future. This review initially discusses the mechanistic advancement into myocardial ischemic injury, focusing on ROS generation (**Figure** **1**). The recent applications of antioxidants and biomaterials for treating myocardial ischemic disease are discussed next. Finally, the current challenges of clinical translation and perspectives in the development of ROS‐scavenging therapeutics are addressed.

## 2 The ROS‐Generated Mechanism in Myocardial IR Injury

Substantial accumulating evidence demonstrates the key role played by oxidative stress in mediating myocardial IR injury. ROS includes a group of molecules derived from oxygen(O_2_) during redox reactions and is the central inducer of oxidative stress. The number of free electrons differentiates the ROS into free radical superoxide (O_2_•^−^), hydroxyl radical (•OH), and non‐radical species such as hydrogen peroxide (H_2_O_2_).^[^ ^20^ ^]^ ROS is more commonly known as the byproduct of aerobic metabolism. Moreover, ROS reversibly oxidizes target proteins in response to internal or external perturbations and thus acts as an indispensable second messenger for cellular activities. However, excessive ROS production results in irreversible lipid peroxidation and DNA damage. It increases the cell membrane permeability and plays a key role in myocardial IR injury. Emerging evidence shows that iron overload and lipid hydroperoxides are closely linked with the occurrence of ferroptosis. Ferroptosis causes around 20% of the total deaths in myocardial ischemic disease. Targeting iron metabolism and ferroptosis in cardiomyocytes is a promising approach for the treatment of ischemic cardiomyopathy. ROS is also responsible for directly causing pyroptosis in cardiomyocytes. Recent studies by Shi et al. prove that ROS induces cardiomyocyte pyroptosis.^[^ ^21^ ^]^ They discovered that H_2_O_2_ or the hypoxia/reoxygenation treatment induces cardiomyocyte pyroptosis mediated by the caspase‐11/GSDMD (Gasdermin D) pathway. Meanwhile, antioxidants (like *N*‐acetyl‐l‐cysteine) significantly reduce GSDMD upregulation and prevent cardiomyocyte death. Furthermore, GSDMD deletion in the cardiomyocytes inactivates pyroptosis in the IR heart, resulting in reduced size of the myocardial infarction. This study brings new insights into ROS‐induced myocardial IR injury and can direct toward a new therapeutic approach for treating ischemic cardiomyopathy. Besides, another study implicates myocardial infarction in activating pyroptosis.^[^ ^22^ ^]^ The therapeutic benefits of a pyroptosis inhibitor (VX‐765) were investigated in mice with myocardial infarction. A remarkable decrease was observed in the infarction area of the heart in the VX‐765 treatment group as compared to the control group. The mechanism of ROS‐induced pyroptosis has helped design strategies that inhibit pyroptosis for ischemic therapy.^[^ ^23^ ^]^ For example, Yue et al. constructed an exosomal microRNA‐182‐5p derived from mesenchymal stem cells for alleviating myocardial IR injury.^[^ ^24^ ^]^ The exosome accumulates in ischemic tissues and inhibits GSDMD‐related pyroptosis, thereby reducing cardiomyocyte death and recovering cardiac function.

### 2.1 Mitochondrial ETC for ROS Generation

Oxidative stress in myocardial IR injury arises from different sources. A leading cause of ROS during myocardial IR injury is the mitochondrial electron transport chain (ETC) complex. Normally, substrate utilization generates reductants like nicotinamide adenine dinucleotide hydride (NADH) and flavin adenine dinucleotide(FADH_2_), which donate electrons to complex I and complex II, forming NAD^+^ and FAD, respectively. The movement of electrons along the ETC ultimately reduces O_2_ into H_2_O at complex IV. O_2_ depletion during ischemia induces a conformational change in complex I, thereby inhibiting its activity. The electron flow is inactivated, and minimal ROS is generated.^[^ ^25^, ^26^, ^27^ ^]^ Nevertheless, the mitochondrial ETC becomes highly inactivated due to the absence of electron recipients. The ROS tends to burst in the reperfusion stage. The ETC remains dysfunctional after reperfusion. Hence, substantial electrons cannot move further to complex IV to fully reduce the re‐entered O_2_ into H_2_O. Instead, they directly bind to the O_2_ and generate massive O_2_•^−^ at complexes I and III.^[^ ^25^, ^28^ ^]^ Complex II has a relatively lower tendency to form O_2_•^−^. A recent study demonstrated that electrons originating from complex II are forced to travel to complex I due to the resistance of moving forward during IR. This movement, reverse electron transport (RET), generates excessive ROS at complex I.^[^ ^29^, ^30^ ^]^ Succinate, the substrate of complex II, accumulates in the ischemic cardiac tissues. RET drives the ROS burst at complex I in response to the rapid oxidation of succinate upon reperfusion. Inhibiting the accumulation of succinate reduces the RET‐mediated ROS production and thus protects against IR injury in vitro and in vivo.^[^ ^29^ ^]^ This aspect further supports the role of complex II in IR injury.

**Figure 2:**
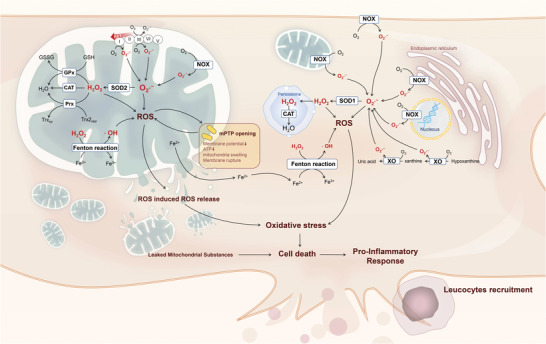
Schematic representation of potential ROS sources in the cardiomyocytes during myocardial IR. Superoxide (O_2_•^−^) anion is largely generated in the mitochondrial ETC at complexes I and III. Particularly, succinate accumulated during ischemia leads to RET, which produces O_2_•^−^ at complex I. Mitochondrial NOX also generates O_2_•^−^. Impaired ROS scavenging systems such as SOD2, CAT, GPx, and Prx/Trx insufficiently eliminate O_2_•^−^ or hydrogen peroxide (H_2_O_2_), leading to ROS accumulation. Excess H_2_O_2_ is converted into a highly reactive hydroxyl radical (•OH) through the Fenton reaction. Mitochondrial ROS, along with the Ca^2+^ overload and restoration of pH, induces the opening of mPTP, further promoting ROS production. Prolonged or excessive opening of the mPTP ruptures the mitochondria, accelerates cell death, and causes ROS generation via an inflammatory response in recruited leucocytes. In the external space of mitochondria, NOX and XO play a major role in ROS production during IR. Moreover, Fe^2+^ released by the ruptured mitochondria initiates the Fenton reaction, generating •OH in the cytosol. Excessive ROS causes oxidative damage to lipids, proteins, and nucleic acids, leading to cell death and exacerbating IR injury.

Mitochondria are the source and the primary target of ROS. Their dense distribution in the cardiomyocytes is responsible for a wide range of damage. This loss from excessive ROS is generated through a self‐amplified pattern, termed ROS‐induced ROS release. Cardiolipin is a critical mitochondrial membrane structural component that interacts with complex enzymes and is damaged by ROS. The loss of cardiolipin sequentially contributes to ETC dysfunction, further promoting ROS generation.^[^ ^28^ ^]^ Furthermore, ROS disrupts cytosolic Ca^2+^ homeostasis and induces mitochondrial Ca^2+^ overload by membrane lipid peroxidation and activation of Ca^2+^ transporters.^[^ ^31^ ^]^ Excessive ROS, mitochondrial Ca^2+^ overload, and restoration of pH during reperfusion open the mitochondrial permeability transition pore (mPTP). This protein dissipates the mitochondrial membrane potential, depletes adenosine triphosphate (ATP), and leaks mitochondrial intermembrane substances, eventually causing cell death.^[^ ^32^ ^]^ The leaked intracellular substances activate the neutrophils and macrophages. This leads to further ROS outbreak from the inflammation cascade and aggravation of myocardial I/R injury.^[^ ^33^, ^34^, ^35^ ^]^ The subsequent leaked intramitochondrial iron contributes to ROS production via the Fenton reaction, which transforms non‐radical H_2_O_2_ into highly reactive •OH.^[^ ^36^ ^]^ A schematic overview of mitochondrial ROS generation is shown in **Figure** **2**.

### 2.2 NOXs for ROS Generation

Nicotinamide adenine dinucleotide phosphate oxidases (NOXs) and xanthine oxidase (XO) are also responsible for the burst of ROS within the cardiomyocytes (Figure 2). The plasma membrane‐associated NOXs deliberately produce ROS to function as the second messenger under cytokine or nutrient stresses.^[^ ^37^ ^]^ NOXs are composed of several isoforms. Cardiomyocytes mainly express NOX1, NOX2, and NOX4, all of which are highly upregulated during IR.^[^ ^38^ ^]^ Systemic knockout of NOX1, NOX2, or both is effective in diminishing ROS production and attenuating the myocardial infarct size after IR.^[^ ^33^, ^39^, ^40^, ^41^ ^]^ NOX4 is the most abundant cardiac NOX isoform with an elusive role. Braunersreuther et al. demonstrated an insufficient benefit of NOX4 knockout in improving IR injury.^[^ ^33^ ^]^ However, Matsushima et al. proved the cardioprotective effects of both systemic and cardiac‐specific knockout of NOX4 in a mice myocardial IR model. Interestingly, NOX2 and NOX4 double knockouts aggravated myocardial IR injury in mice. Thus, NOX4 is also involved in other physiological effects.^[^ ^41^ ^]^ Unlike NOXs that generate ROS specifically, the XO converts purines to uric acids and generates the O_2_•^−^ as a byproduct.^[^ ^42^ ^]^ Elevated levels of XO‐derived ROS are observed in multiple cardiovascular diseases.^[^ ^43^ ^]^ XO is activated by Ca^2+^ overload in cardiomyocytes and produces ROS during myocardial ischemia.^[^ ^44^ ^]^ XO‐derived ROS also impairs mitochondrial function, further increasing ROS production.^[^ ^45^ ^]^ Clinical observational studies demonstrated a negative association of using allopurinol (the specific inhibitor of XO) with the risk of myocardial infarction.^[^ ^46^, ^47^ ^]^ Thus, XO is a key mediator in cardiac disease.

### 2.3 Endothelial NOS for ROS Generation

**Figure 3:**
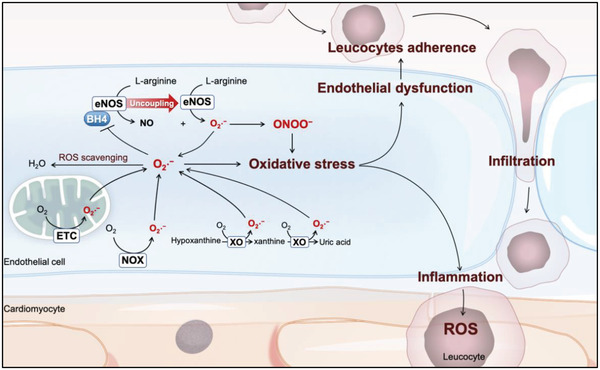
Schematic representation of potential ROS sources in the endothelial cells during myocardial IR. Mitochondrial ETC, cytoplasmic NOX, and XO ubiquitously produce superoxide (O_2_•^−^) in the endothelial cells during ischemia. The eNOS produces nitric oxide (NO) using the cofactor BH4. When O_2_•^−^ oxidizes the BH4 moiety, eNOS undergoes a conformational change termed uncoupling and generates ROS instead of NO. Further, O_2_•^−^ reacts with NO, forming peroxynitrite (ONOO^−^), which is highly oxidative and detrimental. Oxidative stress and low NO availability cause endothelial dysfunction. This phenomenon facilitates the adherence and infiltration of circulating leucocytes, priming the inflammation‐related ROS burst.

Cells other than cardiomyocytes also produce considerable ROS and profoundly affect cardiac function. Endothelial cells (ECs) form the ROS‐generating site during IR and in several cardiovascular diseases that sensitize the heart to IR injury.^[^ ^48^, ^49^ ^]^ ECs critically regulate cardiovascular function through several mechanisms. One such mechanism involves the vasodilating nitric oxide (NO) catalyzed by endothelial nitric oxide synthase (eNOS). However, oxidative stress oxidizes the cofactor tetrahydrobiopterin (BH4) and uncouples eNOS. This event produces additional ROS and halts NO synthesis (**Figure** **3**). Reduced NO induces apoptosis in the endothelial cells and impairs endothelial integrity. Further, the inflammatory cells undergo adhesion and infiltration, inevitably increasing ROS generation through the inflammatory response.^[^ ^50^ ^]^ Overexpression of eNOS restrains the myocardial infarct size in mice after IR. Supplements of BH4 or l‐arginine (the substrate of eNOS) favorably manage to couple the eNOS and ameliorate IR‐induced cardiac damage in animals.^[^ ^51^, ^52^, ^53^, ^54^, ^55^, ^56^, ^57^ ^]^ Increased BH4 levels improve endothelial function in patients with coronary artery disease or hypercholesterolemia.^[^ ^58^, ^59^, ^60^ ^]^ NO shows antioxidative effects within physiological levels.^[^ ^61^, ^62^ ^]^ However, the reaction between NO and O_2_•^−^ may form a detrimental oxidant named peroxynitrite (ONOO^−^).^[^ ^63^ ^]^ Administration of NO donors before or during I/R is cardioprotective in in vivo animal models. However, clinical trials have been misleading.^[^ ^64^ ^]^ The high heterogeneity of patients and an unintended ONOO^−^ formation may together contribute to inconclusive clinical trials. Therefore, NO or its precursor application needs to be monitored precisely after proper adjustment of NO concentration. An inducible NOS (iNOS) is transcriptionally activated in the heart upon IR. iNOS produces aberrant supraphysiological doses of NO, which react with O_2_•^−^ into ONOO^−^. This molecule further aggravates oxidative stress and sharpens myocardial injury.^[^ ^65^, ^66^, ^67^ ^]^

## 3 SOD‐Inspired Strategy for Treating Myocardial Ischemia

Cardiomyocytes prevent oxidative stress by deploying several antioxidant systems that maintain intracellular ROS equilibrium. First, superoxide dismutase (SOD) catalyzes the highly reactive O_2_•^−^ into more stable H_2_O_2_ in situ. The enzyme comprises three isoforms that are located in specific subcellular compartments and require different metallic cofactors for their activity. For example, cytosolic SOD1 and extracellular SOD3 are known as Cu‐ZnSOD for binding copper and zinc, whereas the mitochondrial SOD2 is named MnSOD for using manganese as a cofactor (Figure 2). Increased ROS production upregulates SOD expression at the transcriptional level.^[^ ^68^ ^]^ Nonetheless, SOD is damaged and consumed by excessive ROS through nitration and oxidation of tyrosine residues.^[^ ^69^ ^]^ ROS production is generally more than the antioxidative capacity during myocardial ischemic injury. The antioxidative system plays a critical role. Hence, improving the antioxidant SOD level is a promising therapeutic strategy against the myocardial ischemic disease.^[^ ^70^, ^71^, ^72^ ^]^

SODs are widely used in ischemic myocardial injury therapy. When SOD mimics or a recombinant SOD is administered, a marked reduction is observed in the infarct size following IR in animals.^[^ ^73^, ^74^, ^75^, ^76^ ^]^ Similar observation is demonstrated under genetic overexpression of SOD. Numerous preclinical studies have demonstrated the cardioprotective effects of SOD. Yet, clinical studies do not confirm their effectiveness.^[^ ^77^ ^]^ In early clinical trials, the efficacy of recombinant human SOD was investigated in patients with MI.^[^ ^78^ ^]^ The patient with SOD treatment was invalidated compared with the placebo‐treated group. Currently, the biosafety of PC‐SOD is ongoing at the clinical trial stage (NCT0399573). The enzymatic therapeutics do not work well at the clinical level, probably due to their intrinsic limitations, such as the requirement of high dosage, quick biodegradation, and potential immunogenicity.^[^ ^79^ ^]^ For example, SOD has an extremely short half‐life (≈6 min) after intravenous administration. This aspect severely limits its clinical benefits.^[^ ^80^, ^81^ ^]^ Nanomedicine for SOD delivery can address this challenge. Altshuler et al. loaded nanoparticles with SOD for treating rat myocardial ischemic disease.^[^ ^82^ ^]^ In vivo administration of the nanoparticles significantly enhances SOD retention. Loaded SOD nanoparticles are better at efficiently eliminating ROS and improving cardiomyocyte viability compared to free SOD. In another study, Guo et al. synthesized a metal‐organic framework (MOF) to immobilize SOD (SOD‐ZrMOF) for cardiac injury repair.^[^ ^83^ ^]^ SOD‐ZrMOF was highly biocompatible. In ischemic cardiac tissues, the SOD‐ZrMOF (1 µg/per mouse, intramyocardial injection once) remarkably detoxifies O_2_•^−^, improves cardiac function, and reduces the infarction area. These studies open new avenues for the application of nanomedicine in myocardial ischemic therapy. SOD protects cardiomyocytes by eliminating O_2_•^−^. Nonetheless, the generation of H_2_O_2_ is a persistent challenge before its clinical application. Heme oxygenase‐1 (HO‐1) plays an antioxidant role in myocardial IR injury.^[^ ^84^, ^85^ ^]^ However, upregulation of HO‐1 degrades heme and inevitably increases free iron levels. Heme reacts with H_2_O_2_ and generates highly toxic hydroxyl radicals by the Fenton reaction. Therefore, an appropriate therapeutic benefit of SOD can be achieved by adding catalase (CAT), which detoxifies H_2_O_2_. Multiple enzymatic therapeutics can be explored for treating ischemic‐induced tissue injury considering these enzymes.^[^ ^86^ ^]^

## 4 CAT for Treating Myocardial Ischemic Disease

**Figure 4:**
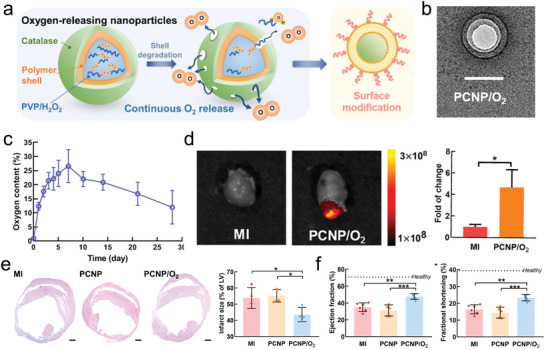
a) Schematic illustration of the function of O_2_‐generating nanoparticles. b) TEM image of an O_2_‐generating nanoparticle. c) The O_2_‐releasing ability of these nanoparticles in 28 days. d) IVIS images and quantification analysis showing the accumulation of nanoparticles in the infarcted areas seven days after surgery. e) H&E staining and quantification analysis for evaluated cardiac remodeling 28 days after surgery. f) Cardiac function of various groups after surgery. Reproduced with permission.^[^ ^93^ ^]^ Copyright 2022, The American Chemical Society.

The next step of ROS scavenging is mediated by the heme‐dependent CAT, which detoxifies H_2_O_2_ into H_2_O and O_2_. CAT is mainly expressed in peroxisomes but is also found in mitochondria.^[^ ^87^ ^]^ CAT is less active in ROS scavenging yet important in protection against cardiac ischemia.^[^ ^88^, ^89^, ^90^ ^]^ Hence, CAT nanomedicine needs to be explored to scavenge ROS and manage myocardial ischemic injury. CAT cannot be directly administered because of its limiting intrinsic properties. CAT also helps in relieving tissue hypoxia. Based on the oxygen‐generating ability, CAT is exploited for ischemia‐related injuries such as cerebral, hepatic, and cardiac ischemia.^[^ ^91^ ^]^ For example, Ding et al. reported improvement in myocardial infarction after applying a CAT hydrogel that scavenges ROS and generates oxygen. In a rat model with myocardial infarction, the group administered CAT hydrogel showed a significantly lower infarct area than other control groups.^[^ ^92^ ^]^ CAT partly alleviates cardiomyocyte hypoxia. But, the oxygen level is deficient and cannot prevent cardiac remodeling. Therefore, the application of O_2_ delivery by CAT has emerged in the fields of ischemic disease. Recently, Guan et al. fabricated an oxygen generation nanoparticle (PCNP/O_2_) using CAT, H_2_O_2_, and a heart‐targeted peptide CSTSMLK (**Figure** **4**).^[^ ^93^ ^]^ The nanoparticle with peptide modification specifically accumulates in the cardiac ischemic site. This design improves the delivery of CAT and reduces the risk of systemic H_2_O_2_ administration. Subsequently, the H_2_O_2_ is converted to H_2_O and O_2_ by CAT. This alleviates cardiac hypoxia, thereby improving cardiomyocyte survival by ATP generation, increasing angiogenesis by endothelial cells, and decreasing cardiac remodeling. This study offers a novel, efficient, and biosafe strategy for O_2_ delivery during myocardial ischemic injury therapy. O_2_ delivery strategies show great potential in alleviating myocardial hypoxia. Different O_2_ delivery nanomaterials are designed to enhance the O_2_ concentration at the ischemic site and to reduce myocardial injury. For example, calcium peroxide (CaO_2_), capable of releasing oxygen, has received extensive attention.^[^ ^94^ ^]^ Shiekh et al. synthesized a bi‐functional cardiac patch with CaO_2_ and antioxidative polyurethane capable of scavenging ROS and releasing O_2_.^[^ ^95^ ^]^ In a rat model with myocardial infarction, the cardiac patch significantly suppressed oxidative injury and resisted remodeling.

HO‐1 plays a critical antioxidant role by catalyzing heme into carbon monoxide (CO), biliverdin, and free iron. Overexpressed HO‐1 is reported to be cardioprotective in mice with myocardial ischemic injury.^[^ ^96^ ^]^ However, deletion of the HO‐1 gene intensifies ischemic cardiac injury and infarction areas.^[^ ^97^ ^]^ Biliverdin is converted into bilirubin by biliverdin reductase, which protects against myocardial IR injury. Bilirubin has antioxidant properties, and it reacts with mitochondrial O_2_•^−^.^[^ ^98^ ^]^ Hence, it has been investigated in various biomedical applications such as colitis, diabetes, and pulmonary fibrosis.^[^ ^99^, ^100^, ^101^ ^]^ However, the challenge of water insolubility needs to be addressed for further clinical application. Various strategies have been employed to overcome this intrinsic deficiency and promote efficient bilirubin delivery. Some examples include the use of PEG‐modified and hyaluronic acid‐modified bilirubin and nanocarrier.^[^ ^102^, ^103^, ^104^, ^105^ ^]^ Recently, Ai et al. self‐assembled PEG‐conjugated bilirubin and synthesized a bilirubin nanoparticle with significantly enhanced solubility and therapeutic benefit of bilirubin.^[^ ^106^ ^]^ Administering bilirubin nanoparticles remarkably reduced the infarction area and cardiac function in a mice ischemic model. Thus, nanomedicine shows great potential in delivering bilirubin efficiently. Additionally, ROS‐responsive bilirubin nanoparticles prepared through self‐assembly improved the therapeutic efficacy and reduced toxicity.^[^ ^103^ ^]^

**Figure 5:**
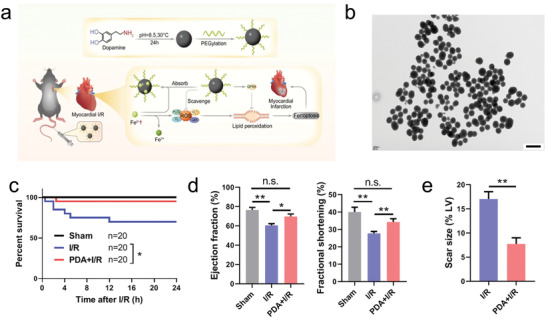
a) Schematic illustration describing the preparation and therapeutic mechanism of PDA (polydopamine). b) Transmission electron microscope image of PDA (Scar bar = 200 nm). c) The survival curves of various groups of mice after different treatments. d) Left ventricular ejection fractions and fractional shortening in different groups at 24 h. e) Quantitative analysis of the scar area seven days after IR. Reproduced with permission.^[^ ^111^ ^]^ Copyright 2021, The American Chemical Society.

Although bilirubin is cardioprotective, HO‐1 upregulation negatively affects ROS production by causing Fe overload, and Fe overload contributes to cardiac injury by inducing ferroptosis.^[^ ^107^, ^108^ ^]^ Ferroptosis is Fe‐dependent cell death associated with Fe‐catalyzed ROS generation and lipid peroxidation. Fang et al. found that HO‐1 overexpression facilitates Fe accumulation in cardiomyocytes that induce myocardial ischemic injury.^[^ ^109^ ^]^ In contrast, administering ferroptosis inhibitors in vivo enhances ischemic cardiomyocyte death and improves cardiac function. Additionally, another study found a positive regulation between that HO‐1‐mediated Ferroptosis and myocardial hypoxia.^[^ ^110^ ^]^ Considering the critical importance of ferroptosis in ischemic injury, targeting ferroptosis can be a promising strategy in myocardial ischemic therapy. For example, polydopamine (PDA) nanoparticles scavenge ROS and inhibit ferroptosis, thus, exerting a cardiomyocyte effect in myocardial ischemic injury (**Figure** **5**).^[^ ^111^ ^]^ The therapeutic benefit of PDA is manifested by the abundant phenolic hydroxyl groups of dopamine, which exhibit high affinity against free Fe and excellent ROS scavenging ability. Injection of PDA into myocardial ischemic mice (20 mg kg^−1^, one intravenous injection) improves cardiac function and reduces the infarction area. Meanwhile, mice with PDA treatment displayed long‐term cardiac benefits than the control mice.

**Figure 6:**
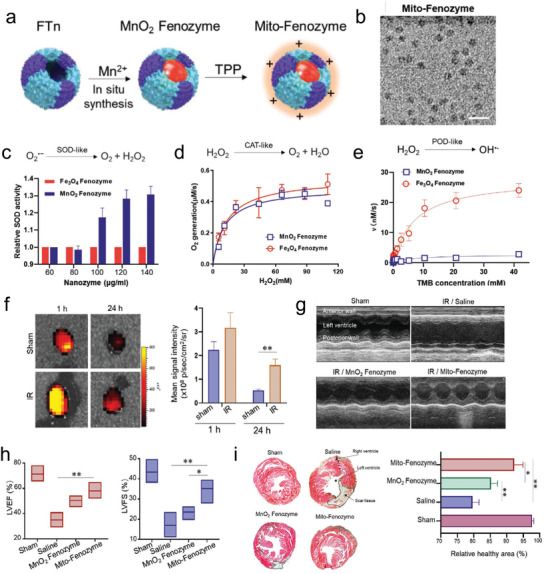
a) Schematic representation describing the fabrication procession of Mito‐Fenozymes. b) TEM image of the Mito‐Fenozymes. The c) SOD‐like, d) CAT‐like, and e) POD‐like abilities of Mito‐Fenozymes. f) Representative in vivo fluorescence imaging and quantification analysis of IR mice after in vivo Mito‐Fenozyme administration. g) Representative echocardiography images and h) Cardiac function analysis of different treatment groups. i) Masson's trichrome staining results of various groups after treatment. Reproduced with permission.^[^ ^113^ ^]^ Copyright 2021, Wiley‐VCH GmbH.

ROS‐scavenging nanozymes with the specific action of mimicking SOD and CAT and protecting against ROS damage are promising targets in treating cardiac ischemic injury.^[^ ^112^ ^]^ Nanozymes possess unique physicochemical properties and bioactivities that provide innovative opportunities to overcome the challenges in myocardial ischemia. For example, numerous metal‐based nanoparticles have inherent enzyme‐mimetic activities, such as CAT and SOD, which detoxify harmful O_2_•^−^ and H_2_O_2_. The chemical state of the surface element governs the catalytic mechanism of these nanozymes. The enzymatic activities of Mn‐based nanozymes are affected by the dominating chemical state. An increased proportion of Mn^3+^/Mn^4+^ reduces the SOD‐like and CAT‐like activities of Mn‐based nanozymes. Zhang et al. constructed a mitochondria‐targeting hybrid nanozyme (Mito‐Fenozymes) using MnO_2_, a ferritin nanocage, and triphenylphosphonium (TPP) for myocardial ischemic therapy (**Figure** **6**).^[^ ^113^ ^]^ In vitro assays demonstrated the high physiochemical stability of nanozymes against harsh conditions such as extremes of temperature and pH. Furthermore, the Mn‐based nanozymes exhibit low POD‐like activity, ensuring their biosafety. The TPP modification confirms the specific accumulation of the hybrid Mn‐based nanozyme in the cardiomyocyte mitochondria. In myocardial ischemic models (2.5 mg kg^−1^, intravenous injection thrice), the nanozymes prevent mitochondrial O_2_•^−^. Consequently, nanozyme administration significantly improves cardiac function and reduces infarction areas than in control groups. Another study reported the use of biometallic CuZn carbon dots (CuZn CDs) with SOD‐ and CAT‐mimicking action in mice myocardial IR injury.^[^ ^114^ ^]^ In a gentle environment (pH 7 and 25 °C), the SOD and CAT action of CuZn CDs is similar to that of natural enzymes. However, the catalytic activities of CuZn CDs are remarkably higher than the natural enzymes during ischemia. An in vivo IR mice model demonstrated the cardioprotective effect of administrating 0.08 mg kg^−1^ (intramyocardial injection, once) of CuZn CDs. Notably, the SOD activity is regulated by the cofactor of the copper element. Cu‐based nanozymes are crucial and are constructed as chaperones for treating ischemia‐related diseases. Recently, Im et al. designed interesting Cu‐deposited ceria nanoparticles (CuCe NPs) that exhibited multienzyme‐mimetic activities and artificial chaperone function.^[^ ^115^ ^]^ The ceria component of CuCe NPs displays SOD‐ and CAT‐like abilities and detoxifies ROS. In the mice MI model, the SOD1 activity is significantly enhanced after intramyocardial administration of CuCe NPs (0.5 mg kg^−1^, around the infarction area, five times). Such reports provide critical motivation for utilizing nanomedicine to regulate ischemia‐related injury.

## 5 GPx‐Inspired Strategy for Treating Myocardial Ischemic Injury

The endogenous selenoprotein family forms an important class of antioxidant systems in the body. It comprises the glutathione peroxidases (GPx), selenoprotein P, selenoprotein T, and thioredoxin reductases (TrxR). Selenoproteins are essential for human health, specifically for their inherent antioxidant properties.^[^ ^116^, ^117^ ^]^ Defective selenoproteins result in the physiological accumulation of ROS, which can disrupt the antioxidant system and cause cell death.^[^ ^118^, ^119^, ^120^ ^]^ Selenoprotein deficiency specifically worsens myocardial ischemic disease by increasing the cardiomyocyte ROS concentration. Jin et al. demonstrated a higher concentration of selenoprotein T in rat cardiac tissues after ischemia; this protein rendered cardioprotection by inhibiting oxidative stress.^[^ ^121^ ^]^ The GPx enzymes are more competitive in ROS clearance than SOD or CAT.^[^ ^122^ ^]^ They degrade H_2_O_2_ and lipid hydroperoxide, the product of lipid peroxidation.^[^ ^123^ ^]^ Four GPx isoforms have been identified so far. GPx1 and GPx4 are mainly expressed in the cardiomyocytes.^[^ ^124^ ^]^ Overexpression of GPx provides remarkable protection to the heart from myocardial ischemic injury.^[^ ^123^, ^125^ ^]^ During ROS detoxification, GPx utilizes two molecules of reduced glutathione (GSH) and forms oxidized glutathione (GSSG). The GSSG is recycled to GSH by glutathione reductase using NADPH as a reductant. Thus, the GSH and nicotinamide adenine dinucleotide phosphate(NADPH) fuel the GPx to exert its antioxidative action (Figure 2). Depletion of GSH or NADPH is a common phenomenon during myocardial ischemia and is associated with myocardial oxidative stress. Supplementing GSH and NADPH or improving their turnover elicits a potent cardioprotective effect during IR.^[^ ^72^, ^126^, ^127^, ^128^, ^129^ ^]^

**Figure 7:**
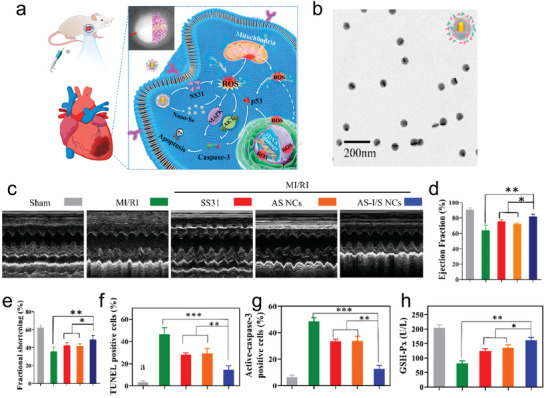
a) Schematic illustration describing the therapeutic mechanism of Au‐Se nanoparticles. b) Transmission electron microscope image of Au‐Se nanoparticles. c) Representative M‐model echocardiography images of different treatment groups. Comparison of d) ejection fraction and e) fractional shortening of various experimental groups. Quantitative analysis of the f) TUNEL‐positive cells, g) active caspase‐3‐positive cells, and h) GSH‐Px content of various experimental groups. Reproduced with permission.^[^ ^134^ ^]^ Copyright 2022, The American Chemical Society.

The levels of selenoproteins correlate positively with the levels of selenium (Se), their active center. A study demonstrated that sodium selenite improves the transcriptional expression of selenoproteins and prevents brain hemorrhage.^[^ ^130^ ^]^ Certain disadvantages need to be addressed despite the successful application of Se in treating various diseases. The balance between drug effectiveness and potential systemic toxicity is one of the primary challenges for the clinical application of Se. Se nanomaterials with high biocompatibility can be exploited to promote the transcriptional regulation of endogenous selenoproteins to restrict ROS‐induced injury.^[^ ^131^, ^132^ ^]^ Tian et al. designed GPx‐mimetic nanomaterials based on the natural structure and function of GPx to prevent brain IR injury.^[^ ^133^ ^]^ Se nanoparticles also alleviate acute kidney injury by activating GPx1 expression.^[^ ^132^ ^]^ In another study, Sun et al. constructed targeted Au‐Se nanoparticles based on the myocardium‐targeted peptide and mitochondrial‐targeted peptide (**Figure** **7**).^[^ ^134^ ^]^ In a mice myocardial ischemic model, in vivo administration of Au‐Se nanoparticles (10 mg kg^−1^, intravenous injection thrice) results in their specific accumulation in the cardiomyocytes and subsequent transfer to the mitochondria. These nanoparticles notably reduced cardiomyocyte apoptosis and oxidative injury by scavenging mitochondrial ROS and inhibiting ROS‐induced caspase‐3 activation. Such studies offer important insights into the application of Se nanomaterials for treating myocardial ischemic injury and may facilitate the development of more advanced therapeutics.

Other nanomaterials, such as Cu nanoparticles, vanadium‐based nanomaterials, and cobalt‐based nanomaterials, with GPx‐mimicking properties, also have garnered increasing interest in biomedicine.^[^ ^135^, ^136^, ^137^, ^138^ ^]^ For instance, an engineered vanadium carbide nanozyme with multienzyme activities (SOD‐, CAT‐, and GPx‐like mimetics) was reported for ischemic stroke treatment.^[^ ^139^ ^]^ Vanadium carbide reduces H_2_O_2_ and oxidizes GSH, similar to GPx. The metal‐ligand coordination in the nanomaterials is an important aspect governing the GPx activity. Recently, Wu et al. investigated the GPx‐like action of different ligands in a vanadium‐based MOF.^[^ ^138^ ^]^ They identified the role of 1,4‐benzene dicarboxylic acid (BDC) ligands with various groups (—F, —Br, —NH_2_, —CH_3_, —OH, and —H) in imparting the GPx‐like activity of vanadium‐based MOF. In BDC, replacement with —F, —Br, —NH_2_ groups exhibit higher GPx‐like activity than using —CH_3_, —OH, and —H groups. In an inflammatory‐related mice model, MOF‐NH_2_ exhibits a strong GPx‐like action that eliminates ROS, thereby benefiting disease therapy. The progress in this field identifies GPx‐mimetic nanomaterials as practical options to treat myocardial IR injury.

## 6 Nitric Oxide‐Inspired Strategy for Treating Myocardial Ischemic Injury

**Figure 8:**
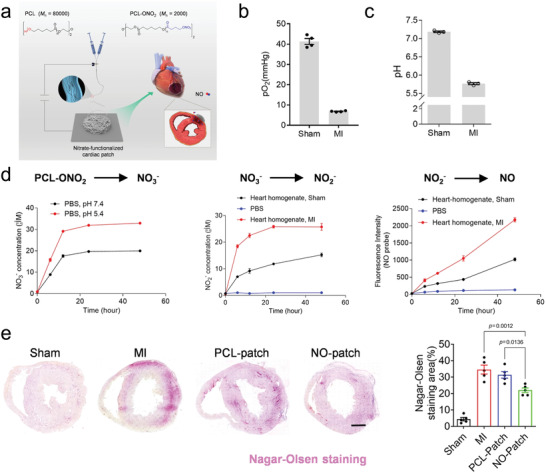
a) Schematic illustration describing the preparation of a cardiac patch to treat myocardial infarction. The b) pO_2_ and c) pH change after myocardial infarction. d) The in vitro NO release from the cardiac patch at various conditions. e) Representative Nagar‐Olsen staining of the infarction heart tissue and quantitative assays of the staining area after myocardial infarction in different groups. Reproduced with permission under the term of CC‐BY license.^[^ ^142^ ^]^ Copyright 2021, the Authors, published by Springer Nature.

Early studies identified the antioxidant action of the vasodilator NO in several mechanisms. For instance, NO directly acts on NOXs, reducing ROS production in neutrophils.^[^ ^140^ ^]^ Decreased endothelial NO level leads to myocardial ischemic injury and remodeling. However, supplementation with exogenous NOS or NO donor in the myocardium reduces oxidative injury and myocardial infarct area and improves cardiac function. Recently, a nanoplatform was loaded with nitric oxide synthase and applied as a carrier to release NO for treating hepatic ischemic therapy.^[^ ^141^ ^]^ Providing NOS supply using nanotechnology is a feasible plan to treat myocardial IR injury. Various NO donor drugs have already been reported to ameliorate myocardial ischemic injury. NO also activates angiogenesis to recover blood supply and thus protects the myocardium against adverse remodeling after infarction. Despite significant progress in developing NO donor drugs, the poor drug tolerance and bioavailability have limited their therapeutic value in clinical trials. These challenges can be addressed by using biomaterials, which achieve sustained therapeutic NO for treating myocardial ischemia and avoid the adverse effects of systemic supply. Zhu et al. constructed a NO‐delivery cardiac patch by covalently binding nitrate for treating myocardial infarction (**Figure** **8**).^[^ ^142^ ^]^ Once inside the infarction tissues, a pH‐dependent degradation process releases the inactive nitrate anion. Subsequently, endogenous enzymes convert the nitrate anion into NO, which effectively enhances cardiac function, decreases infarct size, and mitigates heart remodeling.

Interestingly, ROS overexpression is a risk factor for myocardial ischemia. However, ROS‐responsive NO release system is a practical strategy that enhances drug safety and potency. Currently, intelligent NO‐delivery drugs are being designed using the characteristics of abnormal ROS. Hou et al. reported the construction of the MitoSNOD drug from TPP and diphenylphosphinic chloride for ameliorating myocardial ischemic injury. This drug specifically targets intracellular mitochondria, responds to ROS levels, and efficiently releases NO when triggered by O_2_•^−^.^[^ ^143^ ^]^ MitoSNOD is superior to S‐nitroso‐*N*‐acetylpenicillamine in markedly improving the cardiomyocyte survival rate in a hypoxia H9C2 model. In another study, Hao et al. described a ROS‐responsive hydrogel for in vivo on‐demand NO release.^[^ ^144^ ^]^ The hydrogel releases NO at the ischemic site under ROS stimulation. Consequently, angiogenesis is enhanced, and adverse cardiac remodeling is reduced. The research progress in NO delivery hydrogels depicts promising applications for targeted gas delivery. Noteworthily, biological NO homeostasis is affected by the levels of organic Cu. The major storage of biological Cu occurs via the ceruloplasmin protein. It is synthesized by the liver and benefits cardiac function in ischemic cardiomyopathy.^[^ ^145^, ^146^ ^]^ An artificial copper‐containing protein was designed with a function similar to ceruloplasmin. In a groundbreaking study, Zhang et al. reported biomimetic nanocells by integrating hypoxic stem cells, cell membranes, and copper‐containing ferritin nanocages (Fth‐Cu).^[^ ^147^ ^]^ These artificial nanocells generate NO and enhance angiogenesis by depleting endogenous S‐nitroso thiols. In a mice model of MI, nanocells exhibit a protective ability against ischemic myocardium after intravenous administration (equivalent FTn‐Cu of 1 mg kg^−1^). This investigation gives ideas for constructing biomimetic nanomedicine in the application of ischemic‐related diseases.

## 7 Summary and Outlook

To date, myocardial IR injury remains a large obstacle to the improvements of myocardial infarction followed by interventional treatment. Considering the significant progress in the field of reperfusion research over the past 40 years, a deep understanding has been made of the pathophysiology of myocardial IR injury. The concept that ROS acts as the initial insult and intertwines with other pathological features throughout the myocardial IR injury is now well established and supported by an increasingly large body of evidence. Considering the mitochondrial robustness in the production of ROS, as well as its vulnerability to oxidative stress, we emphatically discussed the origin and functional significance of mitochondrial ROS in the development of myocardial ischemic injury. Compared to the ischemic preconditioning or postconditioning, direct enhancement of endogenous antioxidants is likely more practicable in light of the urgency of timely interventional treatment. Various antioxidant treatments have shown beneficial effects in animal studies, however, the translation into clinical practice has proven to be challenging. One of the major limitations of antioxidant treatments is the biochemical or physiological features. Innovative nanotechnology and nanomedicine strategies have led to the development of advanced nanomedicine for antioxidative therapy. Therefore, we discussed also recent progress in the development of nanomedicine for myocardial ischemic therapy, focus antioxidant therapy has been discussed in the review (**Table** **1**). However, certain critical challenges need to be addressed. 1) An important aspect of clinical therapy using biomaterials is their accumulation at ischemic sites. Modifying drug candidates using nanotechnology is an effective method to overcome the intrinsic limitations of antioxidant enzymes. Nanomedicine thus offers a novel promising strategy against myocardial ischemic injury. However, many reported therapeutic nanoparticles are unable to target the heart and probably get cleared by the liver or kidney. Therefore, the distinctive ability to target the heart or ischemic tissues is a prerequisite. ROS‐responsive biomaterials show great potential in this regard for achieving myocardial ischemic therapy. The local tissue microenvironment governs the responsiveness of the nanomaterials. However, the ROS levels in an ischemic microenvironment are unclear or debatable. Another favorable method to achieve intelligent cargo delivery in myocardial ischemic therapy includes the smart‐responsive strategies induced by external stimuli such as ultrasound, light, and hyperthermia. Additionally, some targeted peptides, smart molecules, and antigens confer a specific ability to the nanomaterials to explicitly select the heart tissue. Thus, targeted nanomedicine is very effective, but fundamental research needs to translate into clinical application. 2) Nanomaterials possessing enzymatic activities (nanozymes) are valuable in myocardial ischemia. However, the size, active component, ligand, synthetic process, and surface structure of the nanozymes affect the enzymatic action, and subsequently, the clinical applications of these nanomaterials. Efficient and selective nanozymes can be constructed when the association between the nanoparticles is clearly understood. The detailed catalytic mechanism of nanozymes should be investigated using multiple experiments, such as theoretical calculations and electron spin resonance. Some GPx‐like nanozymes are used in biomedicine. Nonetheless, reports on their application in myocardial ischemic injury are few. 3) Using artificial chaperones to mediate natural enzymatic activity is an innovative therapeutic strategy for treating an ischemic‐related injury. For example, Cu‐containing nanoparticles function as a cofactor to regulate the homeostasis of the in vivo antioxidant system. The in‐depth understanding of the biological functions of natural enzymes combined with continuous advances in nanoscience can identify additional types of artificial chaperones for ischemic disease therapy. 4) The biosafety and systemic toxicity of nanomaterials should be investigated thoroughly. The use of nanomedicine increases the half‐lives and drug bioavailability in the myocardium. However, long‐term side effects require investigation after in vivo administration. The pharmacokinetics and biodegradation methods of the nanomaterials should be studied in detail for their future clinical application. The toxicity of nanomaterials is dose‐dependent. Hence, the safe dose, toxic dose, and therapeutic window of the medicine need to be determined. The in vivo metabolic translations and mechanisms of nanomaterials after administration are not completely clear. A metabolomic approach can explore the metabolic profile of the nanomaterials and analyze the metabolic changes occurring within the system. In vivo metabolic evaluations are difficult to perform yet very important for clinical practice. 5) Finally, preclinical studies and clinical trials of biomaterials may yield different therapeutic outcomes. The efficacy of these nanomaterials should be systemically explored in big animal models, such as pigs and monkeys, for a better evaluation of their clinical potential. The validity of the nanomaterials should be further optimized and verified before future clinical applications.

**Table 1:** Natural enzymes‐inspired nanomedicine for therapeutics

Nanoplatform	Functionality	Model	Administration approach	Reference
SOD‐ZrMOF	SOD delivery	MI	Intramyocardial injection	[83]
PCNP/O_2_	Oxygen generation	MI	Intravenous injection	[93]
Cardiac patch (CaO_2_)	Oxygen generation	MI	Intramyocardial administration	[95]
PDA	ROS‐scavenging	IR	Intravenous injection	[111]
Mito‐Fenozymes	SOD‐ and CAT‐mimetic	IR	Intravenous injection	[113]
CuZn CDs	SOD‐ and CAT‐mimetic	MI	Intramyocardial injection	[114]
CuCe NPs	SOD‐ and CAT‐mimetic, copper chaperone	MI	Intramyocardial injection	[115]
Au‐Se nanoparticles	ROS‐scavenging	IR	Intravenous injection	[134]
Cardiac patch (NO)	NO delivery	MI	Intramyocardial administration	[142]
Fth‐Cu	NO generation	MI	Intravenous injection	[147]

To summarize, nanomedicine‐based antioxidant therapies are endowed with great potential for treating myocardial ischemia. Currently, nanomedicine for myocardial ischemic therapy is at the initial stage. Continuous efforts are required to advance the clinically available strategies. Addressing the mentioned challenges can further the development of biomaterials and undoubtedly contribute to future clinical translation.

## Conflict of Interest

The authors declare no conflict of interest.
